# Tumorläsion des Pankreas mit unklarer Dignität

**DOI:** 10.1007/s00104-024-02132-8

**Published:** 2024-08-09

**Authors:** Kilian Dossow, Sara Acciuffi, Christine March, Dörthe Jechorek, Roland S. Croner, Frank Meyer, Sara Al-Madhi

**Affiliations:** 1 Klinik für Allgemein‑, Viszeral‑, Gefäß- und Transplantationschirurgie, Otto-von-Guericke-Universität mit Universitätsklinikum Magdeburg, Leipziger Str. 44, 39120 Magdeburg, Deutschland; 2 Institut für Pathologie, Otto-von-Guericke-Universität mit Universitätsklinikum Magdeburg, Leipziger Str. 44, 39120 Magdeburg, Deutschland; 3 Klinik für Radiologie und Nuklearmedizin, Otto-von-Guericke-Universität mit Universitätsklinikum Magdeburg, Leipziger Str. 44, 39120 Magdeburg, Deutschland

## Hintergrund

Laut Zentrum für Krebsregisterdaten des Robert Koch-Institutes erkrankten im Jahr 2020 deutschlandweit etwa 20.230 an Bauchspeicheldrüsenkrebs (Pankreaskarzinom). Für beide Geschlechter liegt die relative 5‑Jahres-Überlebensrate bei etwa 9 %. Damit hat diese maligne Erkrankung der Bauchspeicheldrüse eine der niedrigsten Überlebensraten unter allen Krebserkrankungen. Die Diagnose kann durch unspezifische Symptome oft erst in fortgeschrittenen Stadien gestellt werden, was die Behandlungsoptionen minimiert [28].

Durch die ungünstige Prognose und die anspruchsvolle operative Therapie sollte die Behandlung des Pankreaskarzinoms in zertifizierten Zentren für Bauchspeicheldrüsenkrebs erfolgen. Die unklare Raumforderung des Pankreas stellt eine anspruchsvolle Differenzialdiagnose dar. Neben verschiedenen zystischen Veränderungen sollte immer an ein Malignom gedacht und weitere diagnostische Schritte sowie die Vorstellung in einem Zentrumstumorboard eingeleitet werden [15].

Im Folgenden soll anhand eines interessanten beispielhaften Kasus eine weitere Differenzialdiagnose zur Pankreasraumforderung mittels wissenschaftlichen Fallberichts dargestellt werden.

## Kasuistik

### Anamnese

Ein männlicher 57-jähriger Patient stellte sich über die chirurgische Pankreasspezialsprechstunde vor. Im Rahmen einer Staging-Computertomographie (CT) bei histologisch gesichertem Prostatakarzinom wurde nebenbefundlich eine Raumforderung im Bereich des Pankreas mit unklarer Dignität gefunden.

In der *Eigenanamnese *berichtet der Patient weder über abdominelle Beschwerden noch über B‑Symptomatik. Vor über einem Jahr sei er nach Treppensturz mit stumpfem Bauchtrauma konservativ behandelt worden.

Zusätzlich zeigte sich zu dem erwähnten Prostatakarzinom mit Erstdiagnose 06/2021 und Z. n. offener Prostatektomie eine arterielle Hypertonie und chronischer Nikotin- und Alkoholabusus. Medikamentös wurde der Patient antihypertensiv behandelt (Nebivolol 5 mg 1 × 1, ExForge 5 mg/160 mg/12,5 mg 1 × 1).

In der *klinischen Untersuchung* waren ein guter Allgemein- und normosomer Ernährungszustand zu erheben. Kardiopulmonal war der Patient unauffällig. Grob neurologisch zeigten sich ebenfalls keine Auffälligkeiten. Das Abdomen war weich und präsentierte keine Abwehrspannung, keine Resistenzen, keinen Druckschmerz und eine regelrechte Peristaltik. Im Unterbauch war eine reizlose mediane Laparotomienarbe vorhanden. Skleren- oder Hautikterus lagen nicht vor.

### Initiale Diagnostik

Das *Labor* zum Vorstellungszeitpunkt wies keine Entzündungswerte, keine erhöhten Cholestaseparameter und keine erhöhte Lipase auf. Die GLDH war minimal erhöht. Die *Tumormarker* lagen ebenfalls im Referenzbereich (CA 19-9: < 9 [U/ml], CEA: 2,4 [ng/ml]).

**Abb. 1:**
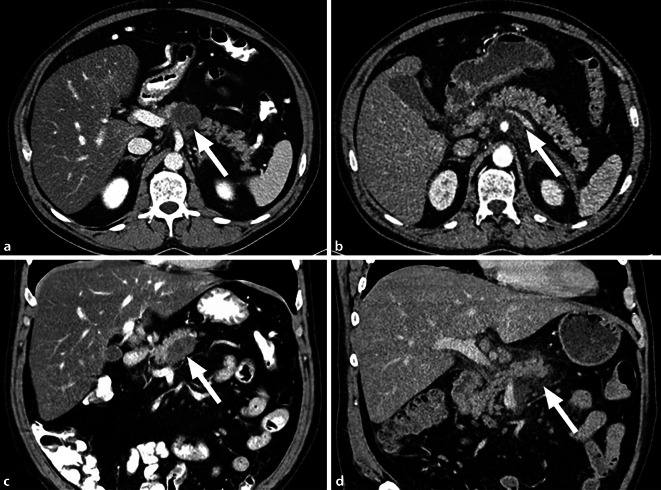
CT-Abdomen: suspekter Pankreaskopftumor. **a,** **b** Zeitpunkt des erhobenen V. a. Raumforderung. **c,** **d** Trauma-CT zum Unfallzeitpunkt ein Jahr zuvor. Jeweils in koronarer und transversaler Ansicht

Eine eingelesene externe *CT des Thorax und Abdomens* zeigte eine Flüssigkeitsformation im Pankreaskorpus, die nicht eindeutig als Raumforderung abzugrenzen war. Eine Cholestase lag nicht vor. Auffällige Lymphknoten oder Filiae wurden ebenfalls nicht beschrieben (Abb. 1).

## Wie lautet Ihre Diagnose?

### Verdachtsdiagnosestellung

Bei Alkohol- und Nikotinabusus, unklarer Pankreasraumforderung und bestehendem Karzinom in der Vorgeschichte (Prostata) wurde der Fall im interdisziplinären Tumorboard mit Malignomverdacht vorgestellt. Zur weiteren Diagnosesicherung sollte eine Endosonographie (EUS) erfolgen.

**Abb. 2:**
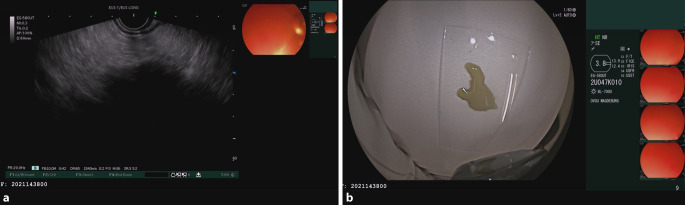
EUS-geführte Punktion: **a** Pankreas unauffällig, wie vorab bereits in der CT zu erkennen – ein semiliquides Areal im Isthmusbereich. **b** Punktat nach transgastraler Punktion mit 19-G-Nadel → Aspiration von gelbgrauer Flüssigkeit – alte Nekrose nach traumatischer Pankreatitis

In der *EUS* zeigte sich ein semiliquides Areal im Isthmusbereich des Pankreas. Das restliche Pankreasparenchym stellt sich normal dar. Der fragliche Befund wurde transgastral punktiert, die aspirierte gelbgrau trübe Flüssigkeit wurde in die Mikrobiologie und Pathologie gesandt (Abb. 2).

**Abb. 3:**
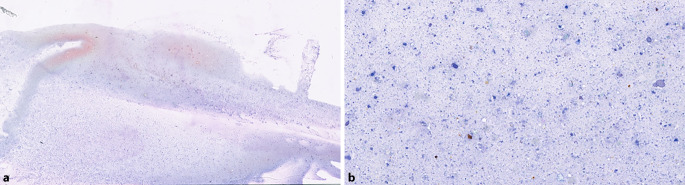
Zytologisches Ausstrichpräparat der Pankreasbiopsie: amorphes Zellmaterial, Zellschutt, Hämoidin – passend zu Residuen einer Nekrose/Zyste → kein Malignitätsnachweis. **a** 10× Vergrößerung, **b** 40× Vergrößerung

Der zytopathologische Befund ergabt eine Pankreasnekrose ohne Malignitätsnachweis (Abb. 3).

### Weitere Therapie und Verlauf

Unter dem Aspekt des zytopathologischen Malignitätsausschlusses erfolgt ein Befundgespräch mit dem Patienten. Hierbei wurde die *Eigenanamnese* erneut evaluiert und Augenmerk auf das vergangene stumpfe Bauchtrauma nach Treppensturz ein Jahr zuvor gelegt. In den damaligen radiologischen Bildern ist eine Pankreaskontusion mit peripankreatischer Flüssigkeitsvermehrung nachzuweisen (Abb. 1). In der erneuten Tumorboardvorstellung wurde der Befund als flüssige Pankreaskorpusformation nach traumatischer Pankreaskontusion und posttraumatischer Pankreatitis gewertet. Bei Beschwerdefreiheit wurde auf eine weitere Therapie verzichtet und eine bildgebende Verlaufskontrolle im Intervall von 3 Monaten per Abdomensonographie und 6 Monaten per CT empfohlen. Hier zeigte sich ein deutlicher Rückgang des Befundes im Pankreas, sodass keine weitere bildgebende Kontrolle empfohlen wurde. Zudem sollte neben der adäquaten Tumornachsorge des Prostatakarzinoms eine Koloskopie im Rahmen der Darmkrebsvorsorge erfolgen. Die Empfehlung zur Nikotin- und Alkoholkarenz wurde dem Patienten ebenfalls übermittelt.

### Definitive Diagnose

Posttraumatische Pankreasnekrose

## Diskussion

In Deutschland weisen etwa 18–20 % aller polytraumatisieren Patienten ein Abdominaltrauma auf [3, 6]. Während bei den häufiger vorliegenden Traumata wie Extremitätenverletzungen (68,9 %) [3] die Diagnosestellung oft bereits durch die Inspektion möglich ist, entstehen 95 % der abdominellen Traumata durch einen stumpfen Verletzungshergang [7, 17] und müssen nicht zwangsläufig offensichtliche Prellmarken oder Verletzungsmuster bieten. Eine unverzügliche und weiterführende Diagnostik ist notwendig und ermöglicht die zeitnahe adäquate Therapie.

Pankreasverletzungen liegen nur in 3,1–11 % aller Abdominaltraumata vor [5–7]. Häufiger betroffen sind andere parenchymatöse Organe wie Milz (32 %) und Leber (20 %) [7]. Begründet werden kann dies durch die topographische Lage im Retroperitoneum [20]. Jedoch darf die Kraftweiterleitung durch die dahinterliegende Wirbelsäule nicht unterschätzt werden. Die Verletzungsschwere des Pankreas teilt sich nach der Klassifikation der American Association for the Surgery of Trauma (AAST) ein und reicht von Kontusion und Kapselrissen bis zur Verletzung des Ductus pancreaticus und der kompletten Ruptur ([18]; Tab. 1). Dadurch stellt das Pankreastrauma trotz der relativen Seltenheit an abdominellen Organverletzungen eine schwere Verletzung mit hoher Morbidität und Mortalität durch mögliche septische Krankheitsverläufe dar [12, 24].

**Tab. 1:** AAST-Klassifikation des Pankreastraumas

Grad	Verletzungstyp	Muster
*Grad I*	Hämatom	Geringe Kontusion ohne Gangbeteiligung
	Lazeration	Oberflächlicher Einriss ohne Gangbeteiligung
*Grad II*	Hämatom	Stärkere Kontusion ohne Gangbeteiligung
	Lazeration	Tieferer Einriss ohne Gangbeteiligung oder Gewebeverlust
*Grad III*	Lazeration	Distale Ruptur oder Parenchymverletzung mit Gangbeteiligung
*Grad IV*	Lazeration	Proximale Ruptur oder Parenchymverletzung mit Beteiligung der Ampulle
*Grad V*	Lazeration	Massive Pankreaskopfdestruktion

Als bildgebende Verfahren zur Diagnosestellung stehen im Schockraum an erster Stelle die FAST(„focused assessment with sonography for trauma“)-Sonographie zur Verfügung, vor allem zum Ausschluss intraabdomineller Flüssigkeit [5]. Bei hämodynamisch instabilen Patienten sollte beim Nachweis abdomineller Flüssigkeit in der Notfallsonographie die Indikation zur sofortigen Laparotomie gestellt werden [17]. Beim stabilen Patienten ist bei jeglichem Verdacht einer Verletzung des Pankreas eine CT des Abdomens mit intravenösem Kontrastmittel durchzuführen [5, 16, 17].

Verletzungen des Pankreas sind durch die retroperitoneale Lage und schwerer abzugrenzender Kapsel (im Vergleich zu Milz und Leber) in der bildgebenden Diagnostik nicht immer suffizient detektierbar [7]. Zudem liegen zum Verletzungszeitpunkt häufig keine oder nur unspezifische abdominelle Beschwerden vor [26]. Komplikationen wie Pankreatitis, Pankreasnekrose, Peritonitis und retroperitonealer Abszess manifestieren sich erst im Verlauf [9]. Werden schwere Verletzungen, insbesondere mit Gangbeteiligung, übersehen, steigt die Morbidität und Letalität durch eine verzögerte adäquate Therapie deutlich [26].

Die Therapie des Pankreastraumas ist abhängig von der Schwere der Verletzung (Tab. 1). AAST-Grad I- bis -II-Verletzungen sind konservativ behandelbar. Beim Auftreten von Hämatomen oder Nekrosen (AAST Grad III) können operative oder endoskopische Ausräumungen sowie Drainageanlagen notwendig sein. Operative Maßnahmen richten sich nach dem Verletzungsmuster mit Gangbeteiligung oder kompletter Organdestruktion und sind je nach Verletzungsmuster notfallmäßig oder im Intervall notwendig [6, 7, 10, 21]. Bei uneindeutiger Gangverletzung in der CT kann eine MRCP zur weiteren Diagnostik zielführend sein [20].

Im vorliegenden Fall wurde die Notfalldiagnostik nach dem beschriebenen Treppensturz mit stumpfem Bauchtrauma ein Jahr vor der Vorstellung in der Pankreasspezialsprechstunde ebenfalls mit eFAST und CT-Diagnostik durchgeführt. Es ist von einer Grad-I- bis -II-Verletzung nach AAST auszugehen, welche eine alleinige konservative Therapie nach sich zog.

Die Überweisung in die Pankreassprechstunde der berichtenden Einrichtung entspricht der Empfehlung, neu aufgetretene Pankreasraumforderungen in einem Zentrum mit interdisziplinärer Expertise in der Behandlung von Tumoren der Bauchspeicheldrüse vorzustellen [15]. Als sichere Risikofaktoren für die Entstehung eines Pankreaskarzinoms gelten unter anderem Nikotin- und Alkoholabusus [28]. Insbesondere unter dem Aspekt der vorhandenen Risikofaktoren des vorgestellten Patienten und der Nebendiagnose eines Prostatakarzinoms war die zeitnahe Vorstellung in einem gastrointestinalen oder hepatobiliären Tumorboard indiziert und erfolgt.

Die beschriebene flüssige Pankreasläsion konnte in der vorliegenden CT nicht eindeutig einer zystischen oder tumorösen Raumforderung zugeordnet werden. Als Differenzialdiagnosen eines Pankreaskarzinoms sind vor allem zystische Veränderungen („cystic pancreatic lesions“, CPL) der Bauchspeicheldrüse zu nennen. Unter neoplastische Zysten fallen unter anderem intraduktal papillär-muzinöse Neoplasien (IPMN), serös-zystische Neoplasien (SCN), muzinös-zystische Neoplasien (MCN) und solide pseudopapilläre Neoplasien (SPN; [13, 27]).

Metastasen anderer Karzinome in der Bauchspeicheldrüse machen unter 2 % aller tumorösen Pankreasläsionen aus [2]. Die Metastasierung ins Pankreas tritt selten auf und hat ein heterogenes Primärtumorspektrum. Neben Nierenzell- und Magenkarzinomen gibt es Daten zu Ursprungstumoren aus Lunge, Kolon, Ovarien, Mammae, Gallengängen, Uterus sowie zu Metastasen von Sarkomen und Melanomen [1, 2, 19, 23, 25, 29]. Die Häufigkeitsverteilung unterscheidet sich in der Literatur stark. Ein „case report“ aus Dänemark berichtet von Pankreasmetastasen bei einem Prostatakarzinom [8], besonders interessant unter der klinischen Befundkonstellation des berichteten Patienten.

Unter dem Aspekt des vorangegangenen Pankreastraumas mit posttraumatischer Pankreatitis besteht zudem die Differenzialdiagnose einer Pseudozyste, die die häufigste Spätfolge konservativ und operativ behandelter Pankreasverletzungen darstellt [5, 7, 11, 14, 22].

Bei limitierter Differenzierung in der durchgeführten CT erfolgte eine EUS. Diese kann komplementär mit besserer Auflösung und Echtzeitbildgebung die Diagnosestellung unterstützen, nicht zuletzt unter der Möglichkeit einer histologischen/zytologischen Sicherung mittels Feinnadelbiopsie (FNA; [4, 13]).

Der Karzinomverdacht des präsentierten Patienten konnte durch die erweiterte EUS-Diagnostik und den zytopathologischen Malignomausschluss nicht bestätigt werden. Nicht zuletzt ergab sich in Kombination mit der neu aufgearbeiteten Anamnese des stumpfen Abdominaltraumas die erklärbare Diagnose einer Pankreasnekrose nach posttraumatischer Pankreatitis bei Pankreaskontusion.

Als erzielbare Erkenntnis aus dem lehrreichen vorgelegten Fall ist abzuleiten, dass

- das Pankreastrauma eine seltene, aber möglicherweise lebensbedrohliche Verletzung darstellt, die besonderer Aufmerksamkeit im Hinblick auf Diagnostik und Therapie des in der Notaufnahme oder im Schockraum arbeitenden Arztes bedarf;
- die unklare Pankreasraumforderung multiple und nicht leicht zu unterscheidende Differenzialdiagnosen hat und eine interdisziplinäre Vorstellung in einem pankreaserfahrenen Zentrum empfohlen ist;
- die Anamnese auf vergangene Diagnosen und Verletzungen weiter einen essenziellen Anteil in der Diagnosefindung inne hat.

*Limitationen* ergeben sich in der seltenen, jedoch nicht unwahrscheinlichen Konstellation des Falles. Dabei ist insbesondere anzuführen, dass es sich „lediglich“ um die Schilderung eines Einzelfalles handelt. Studien, wie in vergleichbar seltenen Fällen sind es zumeist kleine retrospektive Fallserien ohne Kontrollgruppe, existieren für die berichtete klinische Fallkonstellation nicht. Dennoch sind im klinisch-spezifischen Vorgehen des Kasus im Zusammenhang mit der wissenschaftlich relevanten Literatur wertvolle Erfahrungen im diagnosebezogenen Fallmanagement zu eruieren gewesen.

## Schlussfolgerung

Das Abdominaltrauma ist ein nicht selten vorkommendes klinisches Bild in der Notaufnahme und stellt eine Herausforderung für den viszeralchirurgischen Fachkollegen dar. Durch die tiefe retroperitoneale Lage des Pankreas ist dieses ein weniger häufig betroffenes Organ stumpfer Bauchtraumata, verglichen mit anderen parenchymatösen Organen. Die Schwere der Verletzung, insbesondere des Ductus pancreaticus ist verlaufsbestimmend. Der dargestellte Fall zeigt eindrucksvoll die Reichweite von Spätfolgen pankreatischer Verletzungen auf, ebenso die Wichtigkeit einer genauen Anamnese als Ergänzung zur klinischen Untersuchung sowie zur radiologischen und endoskopischen Diagnostik jeder Pankreasraumforderung.

Der wissenschaftlich aufgearbeitete Kasus stellt sowohl für ärztliche Kollegen der Notfallmedizin und Erstbehandlung als auch für onkologisch tätige Internisten und Chirurgen einen interessanten Zusammenhang von Notfall, Diagnostik, Spätfolgen und onkologischer Medizin dar.

## References

[1] Abrams HL, Spiro R, Goldstein N (1950) Metastases in Carcinoma; Analysis of 1000 Autopsied Cases. Cancer 3(1):74–85. 10.1002/1097-0142(1950)3:1 (〈74::aid-cncr2820030111〉3.0.co;2-7) 15405683 10.1002/1097-0142(1950)3:1<74::aid-cncr2820030111>3.0.co;2-7

[2] Bahra M, Jacob D, Langrehr JM, Glanemann M, Schumacher G, Lopez-Hänninen E, Neuhaus P (2008) Metastasen im Pankreas. Chirurg 79(3):241–248. 10.1007/s00104-007-1390-9 17717640 10.1007/s00104-007-1390-9

[3] Bardenheuer M, Obertacke U, Waydhas C, Nast-Kolb D, AG Polytrauma der DGU (2000) Epidemiologie des Schwerverletzten: Eine prospektive Erfassung der präklinischen und klinischen Versorgung. Unfallchirurg 103(5):355–363. 10.1007/s001130050550 10883594 10.1007/s001130050550

[4] Brugge WR (2015) Diagnosis and Management of Cystic Lesions of the Pancreas. J Gastrointest Oncol 6(4):375–388. 10.3978/j.issn.2078-6891.2015.057 26261724 10.3978/j.issn.2078-6891.2015.057 PMC4502158

[5] Coccolini F, Kobayashi L, Kluger Y, Moore EE, Ansaloni L, Biffl W, Leppaniemi A et al (2019) Duodeno-Pancreatic and Extrahepatic Biliary Tree Trauma: WSES-AAST Guidelines. World J Emerg Surg 14(1):56. 10.1186/s13017-019-0278-6 31867050 10.1186/s13017-019-0278-6 PMC6907251

[6] Heuer M, Hussmann B, Lefering R, Taeger G, Kaiser GM, Paul A, Lendemans S, Trauma Registry of the DGU (2011) Pancreatic Injury in 284 Patients with Severe Abdominal Trauma: Outcome, Course, and Treatment Algorithm. Langenbecks Arch Surg 396(7):1067–1076. 10.1007/s00423-011-0836-1 21847623 10.1007/s00423-011-0836-1

[7] Hildebrand P, Hindel C, Roblick UJ, Bruch HP (2007) Abdominaltrauma. Trauma Berufskrankh 9(2):S127–S131. 10.1007/s10039-006-1205-0

[8] Hult M, Sandstrøm H, Wittendorff HE (2015) ‘[Prostate cancer presenting with diffuse infiltration of metastases to the pancreas. Ugeskr Laeg 177(5):V9140491 25650518

[9] Jacobi T, Nagel M, Saeger HD (1997) Verletzungen des Pankreas. Chirurg 68(6):624–629. 10.1007/s001040050242 9324443 10.1007/s001040050242

[10] Knoop M, Vorwerk T (2003) Erfolgreiche Organerhaltung nach kompletter Pankreasruptur und subtotalem Duodenalabriss durch stumpfes Bauchtrauma im Kindesalter: Ein Fallbericht‘. Zentralbl Chir 128(3):236–238. 10.1055/s-2003-38539 12695932 10.1055/s-2003-38539

[11] Koh EY, van Poll D, Goslings JC, Busch OR, Rauws EA, Oomen MW, Besselink MG (2017) Operative Versus Nonoperative Management of Blunt Pancreatic Trauma in Children: A Systematic Review. Pancreas 46(9):1091–1097. 10.1097/MPA.0000000000000916 28902777 10.1097/MPA.0000000000000916

[12] Langwieler TE, Knoefel WT, Izbicki JR (2001) Das Pankreastrauma – Diagnostik und Therapie. Viszeralchirurgie 36(5):328–330. 10.1055/s-2001-17628

[13] Lariño-Noia J, Iglesias-Garcia J, de la Iglesia-Garcia D, Dominguez-Muñoz JE (2018) EUS-FNA in cystic pancreatic lesions: where are we now and where are we headed in the future? Endosc Ultrasound 7(2):102–109. 10.4103/eus.eus_93_17 29667626 10.4103/eus.eus_93_17 PMC5914181

[14] Lewis G, Krige JE, Bornman PC, Terblanche J (1993) Traumatic Pancreatic Pseudocysts. Br J Surg 80(1):89–93. 10.1002/bjs.1800800129 8428304 10.1002/bjs.1800800129

[15] McGuigan A, Kelly P, Turkington RC, Jones C, Coleman HG, McCain RS (2018) Pancreatic Cancer: A Review of Clinical Diagnosis, Epidemiology, Treatment and Outcomes. World J Gastroenterol 24(43):4846–4861. 10.3748/wjg.v24.i43.4846 30487695 10.3748/wjg.v24.i43.4846 PMC6250924

[16] Meyer F, Bruns C (2018) Kapitel 22: Abdominaltrauma. In: Lippert H, Mantke R (Hrsg) Risiken und Komplikationen in der Allgemein- und Viszeralchirurgie. Thieme, Stuttgart, S 255–275 10.1055/b-0037-149115

[17] Mittelstädt A, Weber GF, Grützmann R (2022) Pankreastrauma‘. In: Grützmann R (Hrsg) Referenz Allgemein- und Viszeralchirurgie – Pankreas, 1. Aufl. Thieme, Stuttgart, S 141–149 10.1055/b-006-163242

[18] Moore EE, Cogbill TH, Malangoni MA, Jurkovich GJ, Champion HR, Gennarelli TA, McAninch JW, Pachter HL, Shackford SR, Trafton PG (1990) Organ Injury Scaling, II: Pancreas, Duodenum, Small Bowel, Colon, and Rectum. J Trauma 30((11):1427–1429 2231822

[19] Nakamura E, Shimizu M, Itoh T, Manabe T (2001) Secondary Tumors of the Pancreas: Clinicopathological Study of 103 Autopsy Cases of Japanese Patients. Pathol Intern 51(9):686–690. 10.1046/j.1440-1827.2001.01258.x 10.1046/j.1440-1827.2001.01258.x 11696171

[20] Nieß H, Werner J (2023) Behandlung von Pankreasverletzungen nach stumpfem Bauchtrauma. Die Chir 94(8):675–681. 10.1007/s00104-023-01898-7 10.1007/s00104-023-01898-7 37369739

[21] Patton JH, Lyden SP, Croce MA, Pritchard FE, Minard G, Kudsk KA, Fabian TC (1997) Pancreatic Trauma: A Simplified Management Guideline. J Trauma 43(2):234–239. 10.1097/00005373-199708000-00005 (discussion 239–241) 9291366 10.1097/00005373-199708000-00005

[22] Rabie ME, El Hakeem I, Al Skaini MS, El Hadad A, Jamil S, Shah MT, Obaid M (2014) Pancreatic Pseudocyst or a Cystic Tumor of the Pancreas? Chin J Cancer 33(2):87–95. 10.5732/cjc.012.10296 23958054 10.5732/cjc.012.10296 PMC3935010

[23] Roland CF, van Heerden JA (1989) Nonpancreatic Primary Tumors with Metastasis to the Pancreas. Surg Gyn Obstetr 168(4):345–347 2928909

[24] Rosch R, Schumpelick V, Großner D (2010) Pankreasverletzung‘. In: Schumpelick V, Bleese N, Mommsen U (Hrsg) Kurzlehrbuch Chirurgie, 8. Aufl. Thieme, Stuttgart, S 200–201 10.1055/b-002-33687

[25] Schulz HU, Dörfler K, Lippert H, Bruns C (2014) Das Pankreas als Metastasierungsorgan. Z Gastroenterol. 10.1055/s-0034-1386315 (KC013)24622869

[26] Sharbidre KG, Galgano SJ, Morgan DE (2020) Traumatic Pancreatitis. Abdom Radiol 45(5):1265–1276. 10.1007/s00261-019-02241-7 (New York) 10.1007/s00261-019-02241-7 31576413

[27] Tiemann K, Klöppel G (2020) Sekundäre, tumorartige, zystische und transplantationsbedingte Pankreasveränderungen. In: Tannapfel A, Klöppel G (Hrsg) Pathologie: Leber, Gallenwege und Pankreas, 3. Aufl. Springer, Berlin:, S 691–705 (Pathologie)

[28] Zentrum für Krebsregisterdaten, and Robert Koch Institut (2023) Krebs – Bauchspeicheldrüsenkrebs. https://www.krebsdaten.de/Krebs/DE/Content/Krebsarten/Bauchspeicheldruesenkrebs/bauchspeicheldruesenkrebs_node.html. Zugegriffen: 18. Juli 2024 (18. July 2024)

[29] Z’graggen K, Fernández-del CC, Rattner DW, Sigala H, Warshaw AL (1998) Metastases to the Pancreas and Their Surgical Extirpation. Arch Surg 133(4):413–417. 10.1001/archsurg.133.4.413 (Chicago, Ill.: 1960) 9565122 10.1001/archsurg.133.4.413

